# Spatiotemporal quantification of metastatic tumour cell growth and distribution in lymph nodes by whole-mount tissue 3D imaging

**DOI:** 10.7150/ijbs.72552

**Published:** 2022-06-13

**Authors:** Jun Li, Chun-Jie Xu, Guang-Ang Tian, Qing Li, Dong-Xue Li, Fang Yan, Yao-Qi Zhou, Pei-Qi Huang, Jia-Xuan Xie, Xu Wang, Shu-Heng Jiang, Ya-Hui Wang, Jian Song, Xue-Li Zhang, Shuang-Qin Yi, Li-Peng Hu, Qing Xu, Xiao-Wei Li, Zhi-Gang Zhang

**Affiliations:** 1 State Key Laboratory of Oncogenes and Related Genes, Shanghai Cancer Institute, Ren Ji Hospital, School of Medicine, Shanghai Jiao Tong University, Shanghai, China.; 2 Department of Gastrointestinal Surgery, Renji Hospital, School of Medicine, Shanghai Jiao Tong University, Shanghai, China.; 3 Department of Central Laboratory, Shanghai Chest Hospital, School of Medicine, Shanghai Jiao Tong University, Shanghai, China.; 4 School of Biomedical Engineering, Shanghai Jiao Tong University, Shanghai, China.; 5 Department of Radiation Oncology, Renji Hospital, School of Medicine, Shanghai Jiao Tong University, Shanghai, China.; 6 Department of Frontier Health Sciences, Graduate School of Human Health Sciences, Tokyo Metropolitan University, Japan.

## Abstract

Lymph nodes (LNs) are a common site of metastasis in many solid cancers. Tumour cells can migrate to LNs for further metastatic colonization of distant organs, indicating poor prognosis and requiring different clinical interventions. The histopathological diagnostic methods currently used to detect clinical lymph node metastasis (LNM) have limitations, such as incomplete visualization. To obtain a complete picture of metastatic LNs on the spatial and temporal scales, we used ultimate 3D imaging of solvent-cleared organs (uDISCO) and 3D rapid immunostaining. MC38 cells labelled with EGFP were injected into the left footpads of C57BL/6 mice. Draining lymph nodes (DLNs) harvested from these mice were cleared using the uDISCO method. Metastatic colorectal cancer (CRC) cells in various regions of DLNs from mice at different time points were quantified using 3D imaging of whole-mount tissue. Several stages of tumour cell growth and distribution in LNs were identified: 1) invasion of lymphatic vessels (LVs) and blood vessels (BVs); 2) dispersion outside LVs and BVs for proliferation and expansion; and 3) re-entry into BVs and efferent lymphatic vessels (ELVs) for further distant metastasis. Moreover, these data demonstrated that mouse fibroblast cells (MFCs) could not only promote LNM of tumour cells but also metastasize to LNs together with tumour cells, thus providing a “soil” for tumour cell colonization. In conclusion, 3D imaging of whole-mount tissue and spatiotemporal analysis of LNM may collectively constitute an auxiliary method to improve the accuracy of clinical LNM detection.

## Introduction

Emerging research has indicated that lymph nodes (LNs) are central sites for the aggregation and recirculation of immune cells. Tumour cells always accomplish metastatic colonization of peripheral or distant organs through LNs 1-3. Lymphatic dissemination is a common route of metastasis in solid cancers. Moreover, metastatic tumour cells always acquire driver mutations during colonization of LNs, which suggests that LNs play an important role in the evolution of cancers 4-6. The accuracy of tumour burden quantification and lymph node metastasis (LNM) detection in solid cancers directly affects clinical tumour-node-metastasis (TNM) staging and prognostication for patients with cancer 7, 8. Therefore, the accurate detection and selective removal of LNs harbouring metastatic tumour cells is valuable for cancer diagnosis and therapy.

To date, histopathological assessment has been the most frequently used method to assess clinical samples. Immunohistochemical (IHC) staining is one of the most commonly used methods to detect histopathological changes in clinical tissues from patients 9-13. Tissues collected for histopathological observation must be sectioned for observation of nonsuperficial cellular structures deep in the tissues 14-16. However, traditional histopathological diagnostic methods have limitations in the detection of metastatic tumour cells in LNs. Thus, obtaining accurate spatiotemporal measurements of metastatic tumour cells, including their number, localization and morphology, in intact tissues is difficult.

In recent years, the investigation of complex cells with large projections has been optimally performed in intact tissues. Recently developed tissue clearing methods have overcome obstacles imposed by light scattering 17-19. Among these tissue clearing methods, ultimate 3D imaging of solvent-cleared organs (uDISCO) was developed to reveal and preserve endogenous fluorescence signals over a duration of months while retaining the advantage of size reduction. uDISCO has been reported to allow subcellular imaging in a single scan to reduce the artefacts of physical sectioning and mosaic imaging 14, 20. Currently, uDISCO has been used in various biomedical applications to study the organization of large organ systems. Furthermore, a 3D rapid immunostaining system has been developed to optimize the process of antibody labelling in intact tissues 21. Using this system, the labelling time of millimetre-thick cleared specimens can be shortened from weeks to hours.

In this study, colorectal cancer (CRC)-related LNM was investigated by establishing a mouse footpad model in which CRC cells were injected into the left footpads of mice. The uDISCO method was used to clear and visualize LN tissues. In addition, co-metastasis of CRC cells and fibroblasts was detected by 3D imaging of whole-mount tissue. The accurate spatiotemporal quantification of metastatic tumour cells in LNs by 3D imaging of whole-mount tissue may improve the accuracy of clinical LNM detection and could complement traditional pathological methods for clinical histopathological diagnosis.

## Materials and Methods

### Cell culture

Mouse MC38 cells were purchased from the American Type Culture Collection (ATCC; Manassas, VA, USA). Mouse fibroblast cells (MFCs) were purchased from iCell Bioscience Inc (iCell Bioscience Inc, Shanghai, China). MC38 cells were cultured in Dulbecco's modified Eagle's medium (DMEM) supplemented with 10% (v/v) foetal calf serum (No. FBS500, Moregate, New Zealand) and 1% antibiotics at 37 °C in a humidified incubator with 5% CO_2_. Serum-free solution for cryopreservation of cells (YC0100, Yoche Biotechnology, Shanghai, China) was used in this experiment.

### Transduction of EGFP by an adenoviral vector

The fifth generation of MC38 cells were cultured in Petri dishes. At approximately 60% confluence, the culture medium was replaced with serum-free RPMI-1640 medium or DMEM. MC38 cells were infected with adenovirus-EGFP at a multiplicity of infection (MOI) of 50, 100, 150 or 200. After 24 hours, cells were cultured with complete culture medium containing 10% (v/v) foetal calf serum. After 48 hours, the expression of EGFP in MC38 cells was visualized under an inverted fluorescence microscope (Olympus Corporation, Tokyo, Japan) to evaluate the infection efficiency and determine the optimum MOI.

### Animals

Male C57BL/6J mice (8 weeks old) were purchased from Shanghai Laboratory Animal Center (SLAC; Shanghai, China). Mice were housed and handled according to protocols approved by the East China Normal University Animal Care Commission. All animals received humane care according to the criteria outlined in the “Guide for the Care and Use of Laboratory Animals” prepared by the National Academy of Sciences and published by the National Institutes of Health (NIH).

### Mouse footpad model

A total of 80 mice were housed in compliance with the guidelines of the East China Normal University Animal Care Commission. The mouse model of CRC cell lymphatic metastasis was established by injection of MC38 cells (5×10^4^ cells in 0.05 ml per mouse) into the left footpads of C57BL/6J mice. On days 5, 10, 20 and 30 after tumour cell inoculation, mice in the MC38 cell injection group were sacrificed, and LN tissues were harvested. Some mice developed metastatic tumours in different regional LNs, including the popliteal and inguinal LNs. The DLNs were collected for uDISCO passive clearing and haematoxylin-eosin (HE)/IHC staining to examine potential secondary tumours under a LaVision UltraMicroscope II (LaVision BioTec GmbH, Bielefeld, Germany) or a standard optical microscope.

### Preparation of uDISCO solutions

Dehydrating solutions were prepared by mixing tert-butanol (No. 360538, Sigma, USA) with distilled water at the following concentrations: 30 vol%, 50 vol%, 70 vol%, 80 vol%, 90 vol% and 96 vol%. Before use, 100% tert-butanol was kept above the melting temperature (i.e., between 23 °C and 26 °C). Dichloromethane (DCM; No. 270997, Sigma, USA) was used as a delipidation solution. The refractive index (RI) matching solution was prepared by mixing BABB (benzyl alcohol:benzyl benzoate = 1:2; Nos. 24122 and W213802, respectively; Sigma, USA) with diphenyl ether (DPE) (No. A15791, Alfa Aesar, USA) at a ratio of 4:1 and adding 0.4 vol% vitamin E (No. A17039, Alfa Aesar, USA). The resulting solution was named BABB-D4. The ratio of BABB to DPE can be further adjusted to achieve better preservation of fluorescence and more tissue transparency.

### uDISCO clearing procedure

All steps were performed in a filtered fume hood with gentle shaking by using 5-ml tubes for whole LNs. The tissue samples were protected from light by covering with aluminium foil and were dehydrated by sequential incubation with 4 ml of 30 vol%, 50 vol%, 70 vol%, 80 vol%, 90 vol%, 96 vol% and 100% tert-butanol for 2 hours each at room temperature. Then, these samples were treated by immersion in DCM for 30 minutes at room temperature to remove lipids. Finally, all tissues were incubated in BABB-D for at least 2 hours until the samples became transparent at room temperature.

### 3D rapid immunostaining

The primary antibody was diluted with 5% BSA in electrophoresis buffer (0.1 M boric acid (pH=8.6) containing 0.1% Triton X-100). Electro-enhanced staining was performed with a constant current density of 0.2 mA mm2. The staining of primary antibodies against LYVE-1 (ab33682, Abcam, USA), CD31 (3528S, Cell Signaling Technology, USA) was conducted for a total time of 3 hours, and the direction of the electric field was switched after 1.5 hours. Upon completion of primary antibody staining, the power was switched off, and the sample was allowed to settle in the sample chamber for a few minutes. For destaining of the primary antibody, the sample was placed within a membrane-free labelling chamber in a sample chamber containing fresh electrophoresis buffer. Destaining was conducted at a constant current density of 0.2 mA mm2 for a total time of 2 hours, with reversal of the electric field direction. Secondary antibody staining was performed using exactly the same conditions used for primary antibody staining.

### Light sheet fluorescence microscopy (LSFM) imaging

Cleared whole-mount LNs were imaged using a LaVision UltraMicroscope II (LaVision BioTec GmbH, Bielefeld, Germany) equipped with a white light laser module (NKT SuperK Extreme EXW-12), 6 fixed light sheet-generating lenses, a scientific complementary metal oxide semiconductor (sCMOS) camera (2560 × 2160, voxel size 6.5 µm, Andor Neo), and a 2 × objective (Olympus MVPLAPO) covered with a 6-mm working distance dipping cap. Sixteen-bit optical section images in two channels (excitation at 561 nm and 640 nm) were acquired at a total magnification of 1.26 (N.A. = 0.07) with a Z-step of 5 µm. During image acquisition, the optically cleared samples were immersed in the RI matching solution, i.e., BABB-D4.

### Confocal microscopy imaging

Confocal images were acquired using a Leica SP8 confocal microscope (Leica TCS SP8, Leica Microsystems, Germany) with a 10 × objective (N.A. = 0.4), and a voxel size of 1.136 µm. Three-colour fluorescence images of LVs and BVs were acquired at excitation wavelengths of 568 nm and 647 nm, respectively. The cleared LNs were immersed in the RI matching solution during imaging (as described above). To ensure the specificity of staining, the untreated LNs were set as control. We set the fluorescence intensity of control group as the background, and subtracted the fluorescence intensity of control group from that of experimental group to exclude non-specific staining. To further detect the fluorescence signal of experimental group, we used confocal microscopy to image tumour cells-associated EGFP signal at single-cell level, and observed the basic characteristics such as the shape or size of the tumour cells.

### Image processing and quantification

The 3D multicolour fluorescence images acquired using confocal microscopy were processed for quantitative analysis using Fiji (version 1.52n, NIH). Background subtraction was performed using the rolling ball algorithm implemented in Fiji. Then, a threshold-based algorithm was applied to segment the tumour cells, BVs or LVs in each imaging channel 22, 23. Finally, an in-house program was written in MATLAB (version 2019a, MathWorks) to calculate the total numbers of voxels associated with tumour cells and the proportions of tumour cells within BVs, LVs and LNs.

### Imaris settings

To enable a clear view of MC38 cell and MFC boundaries, the Surface module in Imaris was used to perform threshold-based 3D segmentation. In addition, we filtered out small objects that were smaller than the normal cell volume for more accurate visualization.

### IHC staining

Paraffin-embedded LN tissue slices (5 μm thickness) were deparaffinized and rehydrated for histopathological evaluation 24, 25. For IHC staining, the sections were incubated with 0.3% hydrogen peroxide/phosphate-buffered saline for 30 minutes and blocked with 10% BSA (No. AD0023-100, Sangon, Shanghai, China). The slides were first incubated with the optimal dilutions of antibodies against LYVE-1 (ab33682, Abcam, USA), CD31 (3528S, Cell Signaling Technology, USA) or Ki67 (ab15580, Abcam, USA) at 4 °C overnight and were then labelled with HRP-conjugated anti-mouse (No. 5470S, Cell Signalling, USA) or anti-rabbit (No. ab136817, Abcam, USA) secondary antibodies at room temperature for 1 hour. Then, the sections were treated with DAB substrate solution (No. S21024-2, Thermo, USA) and counterstained with haematoxylin. All sections were observed and imaged with a microscope (Carl Zeiss, Germany).

### Statistical analysis

Data are presented as the means ± standard errors of the mean (SEMs). Statistical analyses were performed using GraphPad Prism 5 for Windows. One-way ANOVA or two-tailed Student's t test was used for comparisons between groups. Values of P < 0.05 were considered statistically significant.

## Results

### 3D imaging of whole-mount draining lymph node (DLN) tissue and quantification of invaded tumour cells

**Figure 1:**
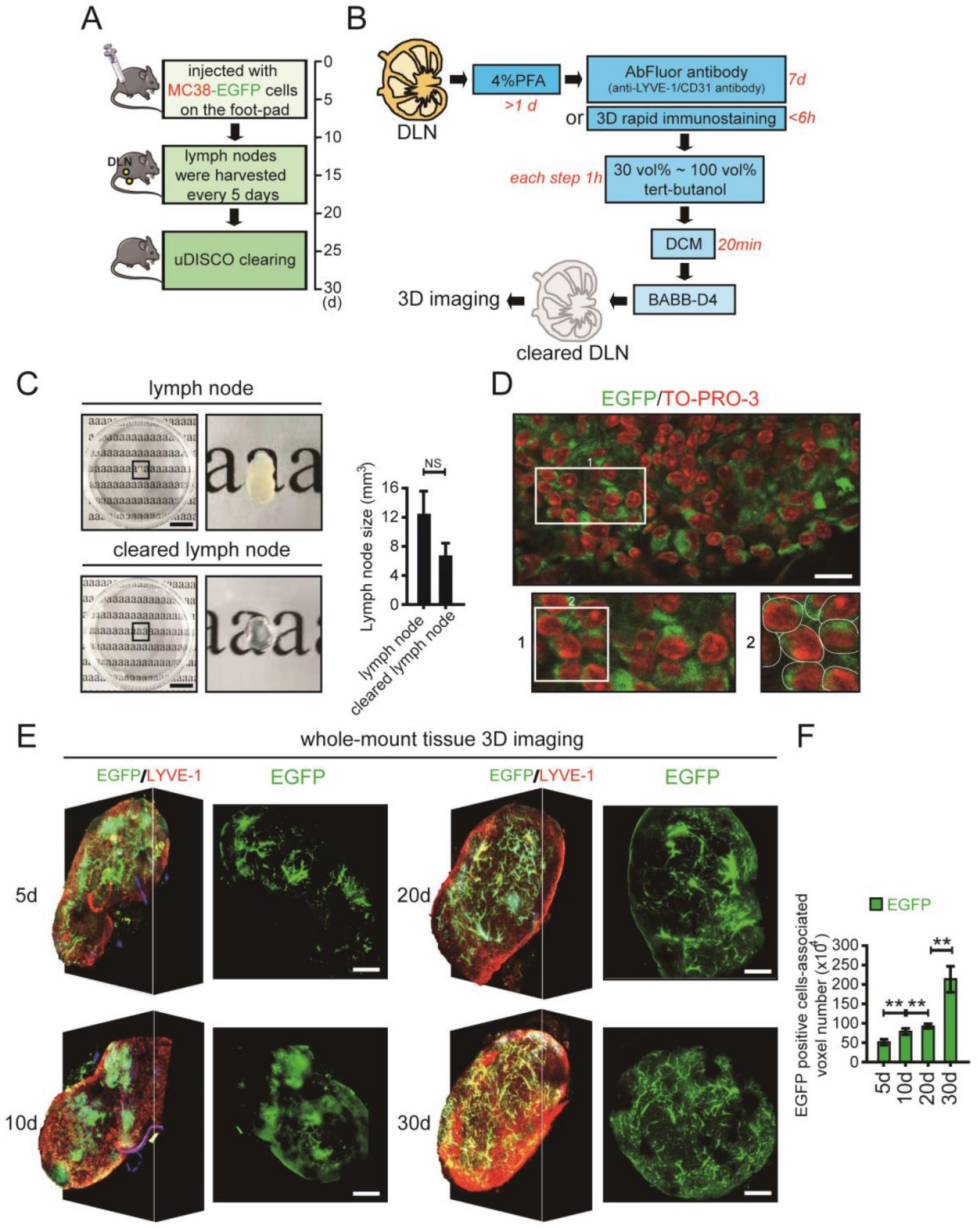
** The whole-mount tissue 3D imaging of DLNs and quantification of tumour cells. A.** The schedule of mouse footpad model construction and the time points of LN tissue collection.** B.** The uDISCO protocols of DLN. **C.** Photos of LN tissues before and after uDISCO clearing. The quantification of the diminution of LN size was shown right. Scale bars: 9.2 mm. **D.** The EGFP positive areas were further observed by a high resolution image in which cell nuclei were counterstained with TO-PRO-3. Scale bars: 20 µm. **E.** 3D images and cross-section photos of DLNs at the time points of 5d, 10d, 20d, and 30d, respectively. Scale bars: 200 µm.** F.** The invaded EGFP-tagged MC38 cells-associated voxel-number in DLNs at the time points of 5d, 10d, 20d, and 30d, quantified by Imaris. At least 6 samples were applied in each group. **P < 0.01; NS: no significant decrease.

The mouse model of LNM was established using a CRC cell line. MC38 cells labelled with enhanced green fluorescent protein (EGFP) were injected into the left footpads of C57BL/6 mice, and the fluorescence intensity was tracked and detected using a Bio-Real *in vivo* imaging system. Based on the fluorescence intensity of EGFP, we chose days 5, 10, 20 and 30 as the main experimental time points (Supplementary Fig. 1 in the Supplementary Material). The DLNs were collected every five days for a total duration of 30 days (Fig. 1A). The harvested DLNs were fixed with 4% paraformaldehyde (PFA) and were then cleared using the uDISCO method (Fig. 1B). The cleared DLNs maintained both the endogenous fluorescence signal and the advantage of size reduction (Fig. 1C). Moreover, the effectiveness of LYVE-1 immunostaining performed by the 3D rapid immunostaining method was similar to that performed by the traditional immunostaining method (Supplementary Fig. 2 in the Supplementary Material). The EGFP positive areas were further observed by a high resolution image in which cell nuclei was counterstained with TO-PRO-3. The merged EGFP and TO-PRO-3 staining indicated the tumour cells (Fig. 1D).

To accurately quantify tumour cells, the cleared DLNs were imaged using confocal microscopy. Because the distribution of tumour cells was dense in some areas and single cells were difficult to distinguish, we used the number of tumour cell-associated voxels instead of the number of tumour cells. By assessing the average diameter (about 11.3 μm) and volume of MC38 cell that was used in our experiment, the voxel (1.13 μm - X axis ×1.13 μm - Y axis × 5.65 μm - Z axis) was used to directly reflect the number of tumour cells (Supplementary Fig. 3A-C in the Supplementary Material). About 105 voxels were set to be approximately equal to 1 MC38 cell by comparing the volume of 1 MC38 cell to 1 voxel (Supplementary Fig. 3D, E in the Supplementary Material). For each group, we calculated the number of tumour cell-associated voxels in the LN image at each time point. After calculation at all time points for a group, the number of tumour cell-associated voxels in the LNs from a particular group of images was obtained. All groups were analysed in the same way to allow intergroup comparisons. The total number of voxels associated with invaded MC38 cells in all DLNs at different time points was quantified using Imaris image processing software (Fig. 1E, Supplementary Movie 1-4 in the Supplementary Material). The number of invaded EGFP-tagged MC38 cells gradually increased in DLNs on days 5, 10, 20 and 30 (Fig. 1F, Supplementary Fig 4 in the Supplementary Material).

### Spatiotemporal distribution of metastatic CRC cells in DLNs

**Figure 2:**
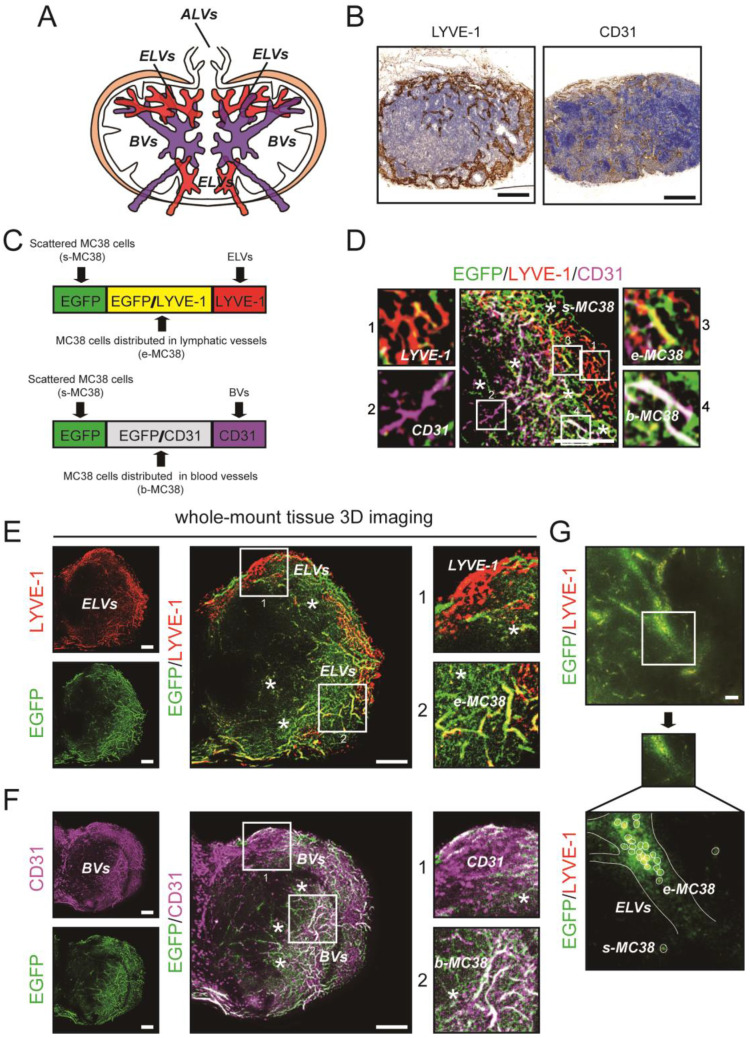
** The immunofluorescent detection of ELVs, BVs, and EGFP-tagged MC38 cells. A.** Distribution of ALVs, ELVs and BVs, including HEVs and arteries in LN. **B.** IHC staining photos of LVs and BVs in LNs. Scale bars: 200 µm. **C.** The immunofluorescent detection of LYVE-1 (LVs) and CD31 (BVs). Colocalization of LYVE1 and EGFP signals indicated MC38 cells distributed in ELVs (e-MC38), colocalization of CD31 and EGFP signals indicated MC38 cells distributed in BVs (b-MC38), and EGFP signal alone indicated MC38 cells scattered outside lymphatic/blood vessels (s-MC38). **D.** Images of EGFP-tagged MC38 cells, LYVE-1 and CD31 in a LN at 5d. LYVE-1: red; CD31: purple; *: MC38 cells scattered outside lymphatic/blood vessels. Scale bars: 100 µm. **E and F.** Cross-section photos of EGFP-tagged MC38 cells, LYVE-1 (**E**) and CD31 (**F**) in a LN at 5d. Scale bars: 100 µm. **G.** Cross-section photos of MC38 cells distributed in ELVs scattered outside lymphatic/blood vessels. Scale bars: 40 µm.

**Figure 3:**
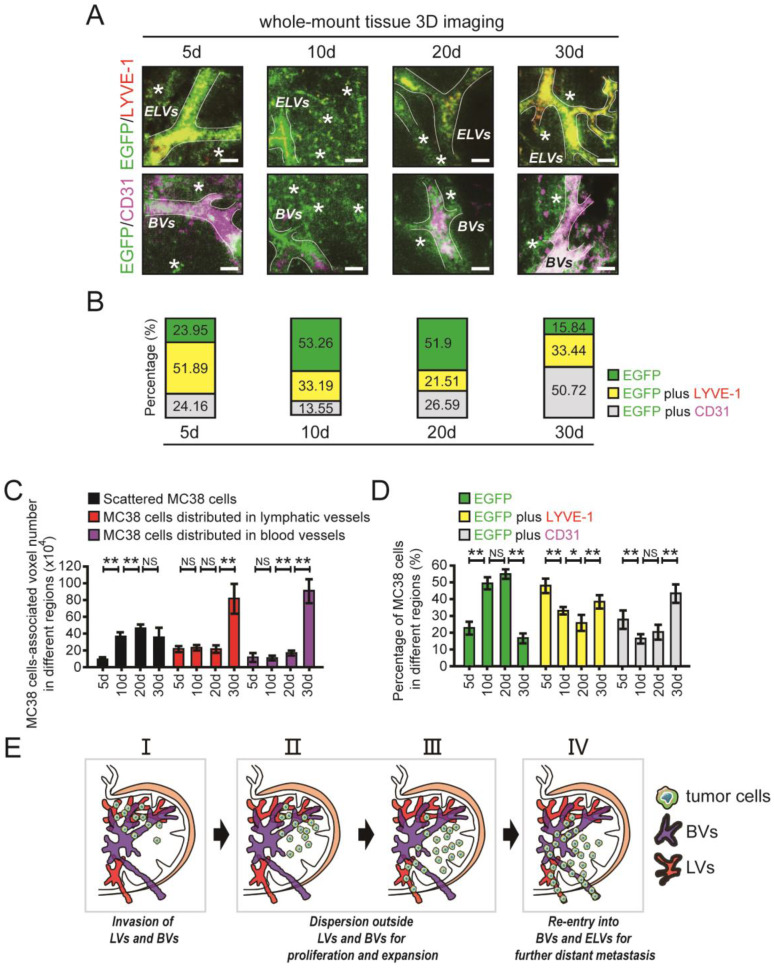
** The spatiotemporal quantification of metastatic CRC cells in DLNs. A and B.** Cross-section photos of EGFP-tagged MC38 cells in the different regions of LNs at 5d, 10d, 20d, and 30d, respectively (**A**). The percentage of MC38 cells distributed in ELVs or BVs / total MC38 cells were shown below (**B**). LYVE-1: red; CD31: purple; *: MC38 cells scattered outside lymphatic/blood vessels. Scale bars: 40 µm. **C.** The voxel-number associated with MC38 cells distributed in ELVs or BVs and the voxel-number associated with MC38 cells scattered outside lymphatic/blood vessels at 5d, 10d, 20d, and 30d, respectively. One-way ANOVA was used for comparison between groups. At least 6 samples were applied in each group. **D.** The ratio of MC38 cells distributed in ELVs or BVs / total MC38 cells and the ratio of MC38 cells scattered outside lymphatic/blood vessels / total MC38 cells at 5d, 10d, 20d, and 30d, respectively. One-way ANOVA was used for comparison between groups. At least 6 samples were applied in each group. **E.** The metastatic tumour cells were found mainly invaded in ELVs and BVs in the early stages of LNM, then most of them moved and scattered outside lymphatic/blood vessels in LNs for proliferation and expansion, and at last reaggregated in BVs and ELVs to support metastasis. *P < 0.05; **P < 0.01; NS: no significant difference.

Previous studies have reported the distribution of different lymphatic vessels (LVs) and blood vessels (BVs) in LNs 26; these LVs include afferent lymphatic vessels (ALVs), efferent lymphatic vessels (ELVs), high endothelial venules (HEVs) and arteries (Fig. 2A). Here, we confirmed the distribution of LVs and BVs in DLNs by IHC staining (Fig. 2B). Then, using 3D imaging of whole-mount tissue, whole LN tissues were immunostained with antibodies against LYVE-1 (for LVs) and CD31 (for BVs). Colocalization of the LYVE1 and EGFP signals indicated MC38 cells distributed in ELVs (e-MC38 cells), colocalization of the CD31 and EGFP signals indicated MC38 cells distributed in BVs (b-MC38 cells), and the EGFP signal alone indicated MC38 cells scattered outside LVs/BVs (s-MC38 cells) (Fig. 2C-F). MC38 cells and the associated number of voxels were visualized and analysed by using Imaris (Fig. 2G, Fig. 3C, D).

After uDISCO clearing, the distribution of EGFP-tagged MC38 cells in the different regions of DLNs was evaluated by 3D imaging (Fig. 3A, B). We then quantified the number of tumour cell-associated voxels in different regions of DLNs. The number of voxels associated with MC38 cells distributed in ELVs or BVs was unchanged between days 5 and 20 but increased significantly from day 20 to day 30. In addition, the number of voxels associated with MC38 cells scattered outside LVs/BVs increased significantly from day 5 to day 10. However, no changes were observed in the number of voxels associated with scattered tumour cells at time point beyond day 10 (Fig. 3C).

Interestingly, a relatively high percentage of MC38 cells were characterized as e-MC38 cells (51.89%) or b-MC38 cells (24.16%) at the early time point of day 5. On days 10 and 20, the e-MC38/total MC38 cell ratio was decreased (33.19% on day 10 and 21.51% on day 20). Similar changes were also observed in the b-MC38/total MC38 cell ratio on day 10 (13.55%) and day 20 (26.59%). At the last time point (day 30), the e-MC38/total MC38 cell ratio (33.44%) and the b-MC38/total MC38 cell ratio (50.72%) were significantly increased. However, the opposite trend was observed for s-MC38 cells: the s-MC38/total MC38 cell ratio was relatively low (23.95%) on day 5, subsequently increased to 53.26% (day 10) and 51.90% (day 20), and finally decreased to 15.84% (day 30) (Fig. 3A and B). At least 6 individual samples per group were evaluated, statistical results are shown in Fig. 3D. Additionally, CD4 cells, CD8 cells (T lymphocytes) and CD19 cells (B lymphocytes) were found distributing around the perivascular area, while ER-TR7 positive cells (fibroblastic reticulum cells, FRCs) and BVs showed cross distribution at the time point of day 20, in which the number of CD4 cells is much more than that of CD8 or CD19 cells (Supplementary Fig. 5 in the Supplementary Material).

The distribution of metastatic tumour cells was determined based on the statistical results. Metastatic tumour cells invaded mainly ELVs and BVs in the early stage of LNM. Then, most of these cells moved and scattered outside LVs or BVs for proliferation and expansion. Finally, tumour cells reaggregated in BVs and ELVs to support further metastasis (Fig. 3E). We also showed that spatiotemporal information for metastatic tumour cells in LNs can be accurately obtained using 3D imaging of whole-mount tissue.

### Proliferation and expansion of metastatic CRC cells in DLNs

**Figure 4:**
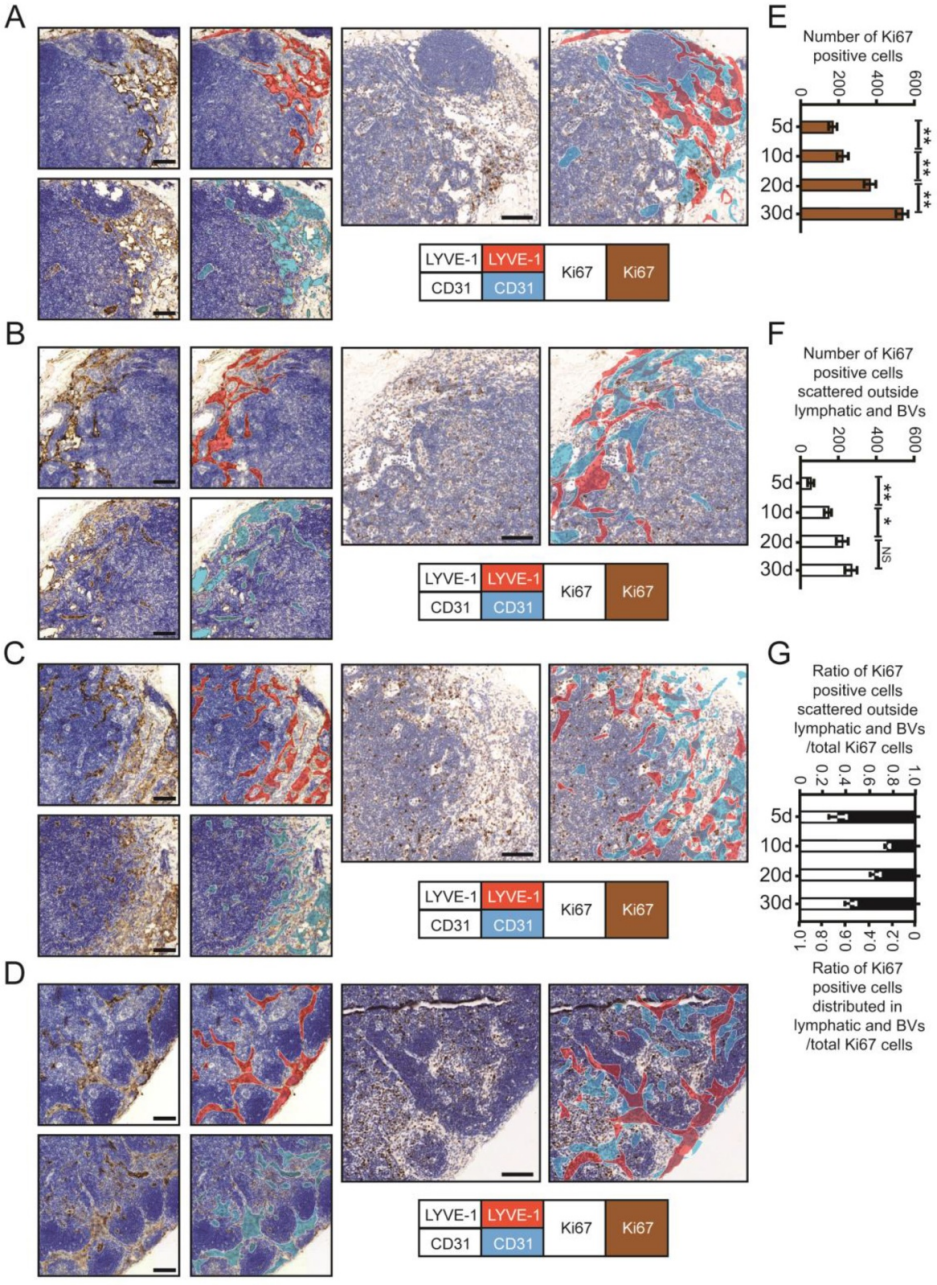
** The proliferation of metastatic CRC cells in DLNs. A-D.** IHC staining photos of Ki67, CD31 and LYVE-1 in DLNs at 5d (**A**), 10d (**B**), 20d (**C**) and 30d (**D**). Scale bars: 100 µm. **E and F.** The statistical results of total Ki67 (**E**) and scattered Ki67 (**F**) (the tumour cells distributed outside lymphatic/blood vessels) positive cells at 5d, 10d, 20d, and 30d, respectively. All experiments were repeated at least 3 times. **G.** The ratio of Ki67 positive cells (scattered outside lymphatic/blood vessels) / total Ki67 cells and Ki67 positive cells (distributed in lymphatic/blood vessels) / total Ki67 cells at 5d, 10d, 20d, and 30d, respectively. *P < 0.05; **P < 0.01; NS: no significant difference.

To further confirm the outgrowth of MC38 cells in different LN regions, we used IHC staining for Ki67 to evaluate the proliferation of metastatic CRC cells in DLNs (Fig. 4A-D). LYVE-1-positive areas (i.e., LVs) were stained red, and CD31-positive areas (i.e., BVs) were stained purple. Then, we superimposed the red and purple areas on the third serial section to identify metastatic tumour cells distributed within or outside LVs/BVs. Total Ki67 (t-Ki67)-positive cells were characterized as tumour cells distributed within the whole LN, and scattered Ki67 (s-Ki67)-positive cells were characterized as tumour cells distributed outside LVs/BVs.

By calculating the number of proliferating tumour cells in different regions, we found that the numbers of both t-Ki67-positive cells and s-Ki67-positive cells increased with time. However, the rate at which the number of s-Ki67-positive cells increased over time decreased between days 20 and 30, while the number of Ki67 cells distributed in LVs and BVs increased significantly during this final time period (Fig. 4E-G). Collectively, the above data further confirmed the spatiotemporal information obtained from 3D imaging of whole-mount tissue.

### Co-metastasis of CRC cells and mouse fibroblast cells (MFCs) in DLNs

**Figure 5:**
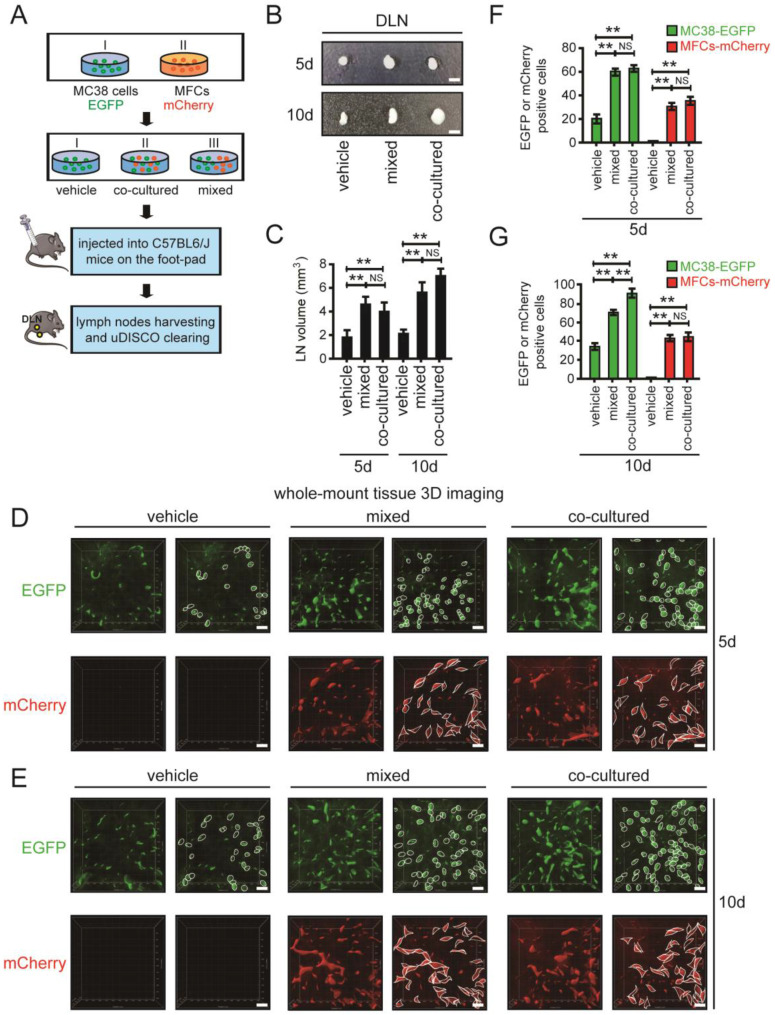
** The co-metastasis of CRC cells and MFCs in DLNs. A.** The schedule of mouse footpad injection. The mice were injected with co-cultured or mixed MC38 cells tagged with EGFP and MFCs tagged with mCherry. MC38 cells alone was set as the vehicle group. **B and C.** Photos of DLNs obtained from mice at 5d and 10d (**B**). The statistical results were shown below (**C**). **D and E.** 3D images of EGFP-tagged MC38 cells and mCherry-tagged MFCs in cleared DLNs at 5d (**D**) and 10d (**E**). Scale bars: 60 µm. A randomly selected 3D image (500 µm - X axis × 500 µm - Y axis × 100 µm - Z axis) was taken out and shown here. The number of MC38 cells or MFCs were quantified according to the 3D images. Thresholds in these images were set as: the surface modules with the size > 10 µm were considered to be MC38 cells (**D**) and surface modules with the size > 15 µm were considered to be MFCs (**E**). **F and G.** The statistical results of the 3D images of EGFP-tagged MC38 cells and mCherry-tagged MFCs in cleared DLNs at 5d (**F**) and 10d (**G**). Scale bars: 10 µm. All experiments were repeated at least three times. **P < 0.01; NS: no significant difference.

Recent reports have indicated that fibroblasts provide a “soil” for metastatic tumour cells 27. We therefore investigated the effects of fibroblasts on CRC cells that disseminated to LN tissues by using 3D imaging of whole-mount tissue. MC38 cells labelled with EGFP and MFCs labelled with mCherry were cocultured or mixed together and divided into three groups: vehicle, coculture and mixed. In the coculture group, MC38 cells and MFCs were mixed together, incubated for 24 hours and injected into the left footpads of mice. In the mixed group, MC38 cells and MFCs were mixed together immediately prior to injection into the left footpads of mice (Fig. 5A).

DLNs were harvested and analysed after 5 days and 10 days. DLNs obtained from the mixed and coculture groups had significantly larger volumes than those in the vehicle group on days 5 and 10 (Fig. 5B, C). DLNs were cleared using uDISCO to analyse infiltrating tumour cells. On days 5 and 10, the number of metastatic CRC cells was significantly higher in the mixed and coculture groups than in the vehicle group, suggesting that MFCs can promote the metastasis of CRC cells (Fig. 5D-G). In addition, MFCs were found to be scattered in LNs on days 5 and 10, where they grew together with CRC cells. These metastatic MFCs in LN tissues provide a “soil” for CRC cells, thus supporting the metastasis and growth of CRC cells in LNs (Fig. 5D-G).

### 3D imaging of whole-mount mesenteric LN tissues from clinical CRC patients

**Figure 6:**
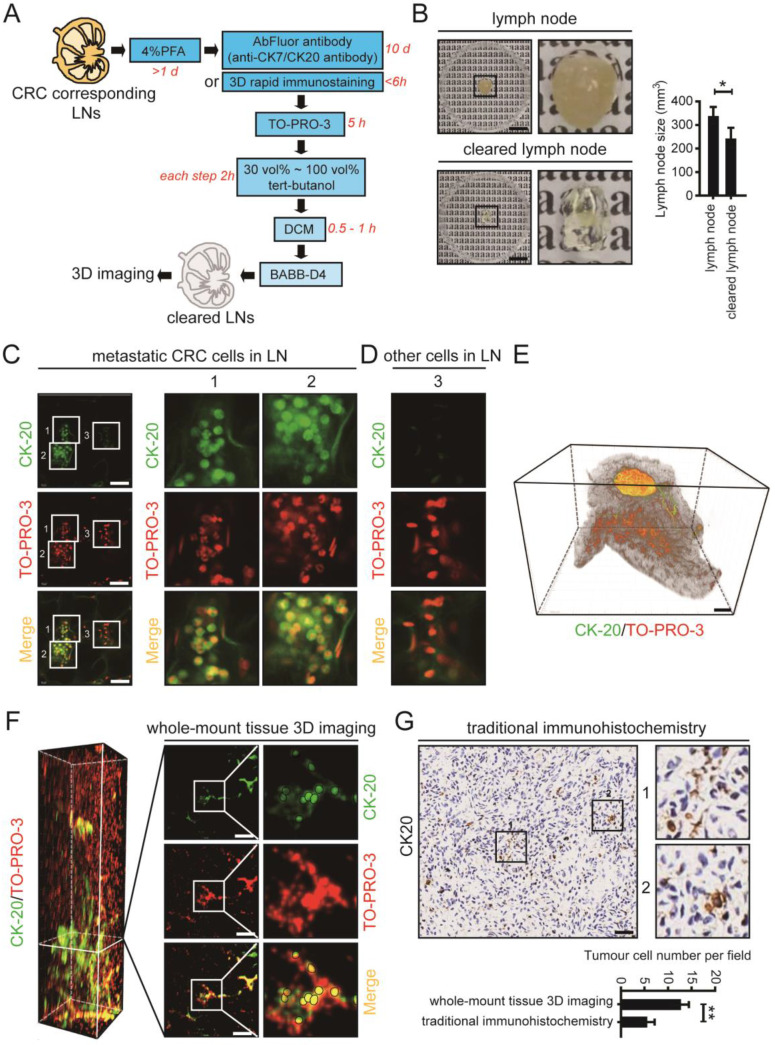
** The whole-mount tissue 3D imaging of mesenteric LN tissues obtained from clinical CRC patients. A.** The uDISCO protocols of clinical CRC corresponding mesenteric LN tissues. **B.** Photos of mesenteric LNs before and after uDISCO clearing. The quantification of the diminution of LN size was shown right. Scale bars: 9.2 mm. **C and D.** Photos of metastatic CRC cells and other cells detected by laser confocal microscope. Scale bars: 50 µm. **E.** 3D imaging of a LN tissue which was collected from a 70-year old man. Scale bars: 800 µm. **F.** Cross-section photos of invaded CRC cells in above LN tissue. Scale bars: 100 µm. **G.** The immunohistochemical staining of the LN used in 3D imaging. Similar locations were selected for the fields (162.5 µm × 162.5 µm) of immunohistochemical staining. Scale bars: 50 µm. The statistical result of tumour cell number per field analyzed by whole-mount tissue 3D imaging or traditional immunohistochemistry was shown below. *P < 0.05; **P < 0.01.

For application of 3D imaging of whole-mount tissue in clinical research, mesenteric LN tissues were collected from CRC patients according to a standard procedure, fixed with 4% PFA, and cleared using uDISCO (Fig. 6A). The cleared LN tissue collected from a 70-year-old man retained the advantage of size reduction (Fig. 6B). After immunostaining with an antibody against CK-20 (to label CRC cells) and the nuclear dye TO-PRO-3 (to label nuclei), we visualized the cleared LNs by laser scanning confocal microscopy. CRC cells labelled with CK-20 were observed and are shown in fields 1 and 2, and unstained cells are shown in field 3 (Fig. 6C, D).

3D imaging of whole-mount tissue was used in conjunction with Imaris 9.0 software to image and process the LN tissue. A reconstructed 3D image of the whole LN was generated (Fig. 6E). Invasion of CRC cells was clearly observed in the LN cross-section (Fig. 6F). Moreover, we collected the same LN tissues which have been used in whole-mount tissue 3D imaging and analyzed it by traditional histopathological methods. Similar locations were selected for the fields of immunohistochemical staining. By comparing the different imaging effects in the two methods, we found that the metastatic tumour cells could be much easier detected using whole-mount tissue 3D imaging (Fig. 6G). Thus, 3D imaging of whole-mount tissue may be used in combination with traditional histopathological methods to improve the accuracy of clinical LNM detection and facilitate clinical diagnosis.

## Discussion

LNs are complex organs that harbour multiple specialized stromal, myeloid, and lymphoid cells. LNs are composed of well-developed smooth muscles under a capsular layer, and lymph flowing in from the ALVs is squeezed out by the capsular smooth muscles into the ELVs. Under physiological conditions, the valves of ALVs and ELVs prevent retrograde flow 28, 29. Previous studies have indicated that tumour cells metastasize to LNs by reconstructing the local immune environment and transforming immune cells, but spatiotemporal changes in tumour cells in whole-mount LNs have not been reported 2, 3, 30. In this study, we used 3D imaging of whole-mount tissue to accurately quantify the number of metastatic CRC cells in DLNs of mice at different time points and in different LN regions. The traditional immunostaining method used in uDISCO clearing requires 1 to 2 weeks for antibody labelling. Here, a 3D rapid immunostaining system was used to shorten the antibody labelling time of intact LNs from weeks to hours during the process of uDISCO. We found that tumour cells enter LNs through ALVs but then proliferate outside LVs and BVs, potentially due to the limited space within these vessels. After the tumour cells in LNs have proliferated to generate a certain number of cells, most of these cells migrate back into BVs and ELVs to facilitate their further distant metastasis. By 3D imaging of whole-mount tissue, we can obtain both spatial and temporal information regarding the distribution of metastatic tumour cells in LNs, providing a theoretical basis for further understanding the biological characteristics of metastatic tumour cells in LNs.

Currently, clinical detection of CRC-related LNs relies mainly on traditional histopathological methods. Intestinal planar and distal LNs are detected by observing CK7, CK20, CDX2 or other markers 31-33. Because these histopathological methods rely on imaging in two-dimensional tissue sections, the information obtained is limited. In a recent study, over 280,000 tissue slices of an entire murine LN were imaged by an extended-volume imaging system to visualize the conduit network in the whole tissue 34. In this study, we aimed to use a simpler method to achieve comprehensive visualization of whole LN tissue and to effectively obtain the spatial and temporal information for metastatic tumour cells in LNs. By using 3D imaging of whole-mount tissue and a 3D rapid immunostaining system for intact tissues, we obtained spatiotemporal information for metastatic tumour cells in LNs and reduced the total time required for the clearing and staining process to 3 days. In the future, this method may play an auxiliary role in the clinical detection of solid cancer-related LNM.

Recent reports have indicated that tumour cells metastasize to LNs very early in cancer progression. During tumour cell-related LNM, tumour cells acquire driver mutations, while stromal and immune cells respond to promote the formation of a “premetastatic niche” in LNs, which creates a tumour microenvironment supporting the colonization and growth of tumour cells in LNs 4, 6. Siglec1-positive subcapsular sinus macrophages have been reported to provide a “soil” for LNM of melanoma cells 6. Here, we observed the co-metastasis of fibroblasts and tumour cells in LNs by 3D imaging of whole-mount tissue. Interestingly, MFCs not only were able to promote tumour cell LNM but also could metastasize to LNs together with tumour cells, thus providing a “soil” for tumour cell colonization. In the future, we will further identify the key molecules that play important roles in the regulatory effects of MFCs on the metastasis and colonization of tumour cells and determine the potential underlying mechanism. 3D imaging was used to reveal this process and may constitute a new method to study the co-metastasis of tumour and stromal cells.

In conclusion, 3D imaging of whole-mount tissue can provide accurate spatiotemporal quantification of metastatic tumour cells in LNs, possibly complementing traditional histopathological methods of clinical diagnosis and affecting clinical TNM staging for patients with cancer. In the future, we may further optimize and combine 3D imaging of whole-mount tissue, 3D rapid immunostaining and spatiotemporal quantification as an auxiliary method to improve the accuracy of clinical LNM detection.

## Supplementary Material

Supplementary figures and movie legends.

Supplementary movie 1.

Supplementary movie 2.

Supplementary movie 3.

Supplementary movie 4.
