# Supplementary material for: Spatiotemporal quantification of metastatic tumour cell growth and distribution in lymph nodes by whole-mount tissue 3D imaging
Source: Int J Biol Sci. 2022 Jun 13;18(10):3993–4005. doi: 10.7150/ijbs.72552 (PMC9274482; doi:10.7150/ijbs.72552)
Supplement: Supplementary file 1 — Supplementary figures and movie legends. [file ijbsv18p3993s1.pdf]

Supplementary Material

Supplementary Movie 1-4. The whole-mount tissue 3D video of DLNs.

The whole-mount tissue 3D video of DLNs at the time points of 5 d (Movie 1), 10 d (Movie 2), 20 d (Movie 3) and 30 d (Movie 4). Based on the analyses of Imaris, the invaded MC38 cells-associated pixel number in the whole DLNs at different time points was quantified.

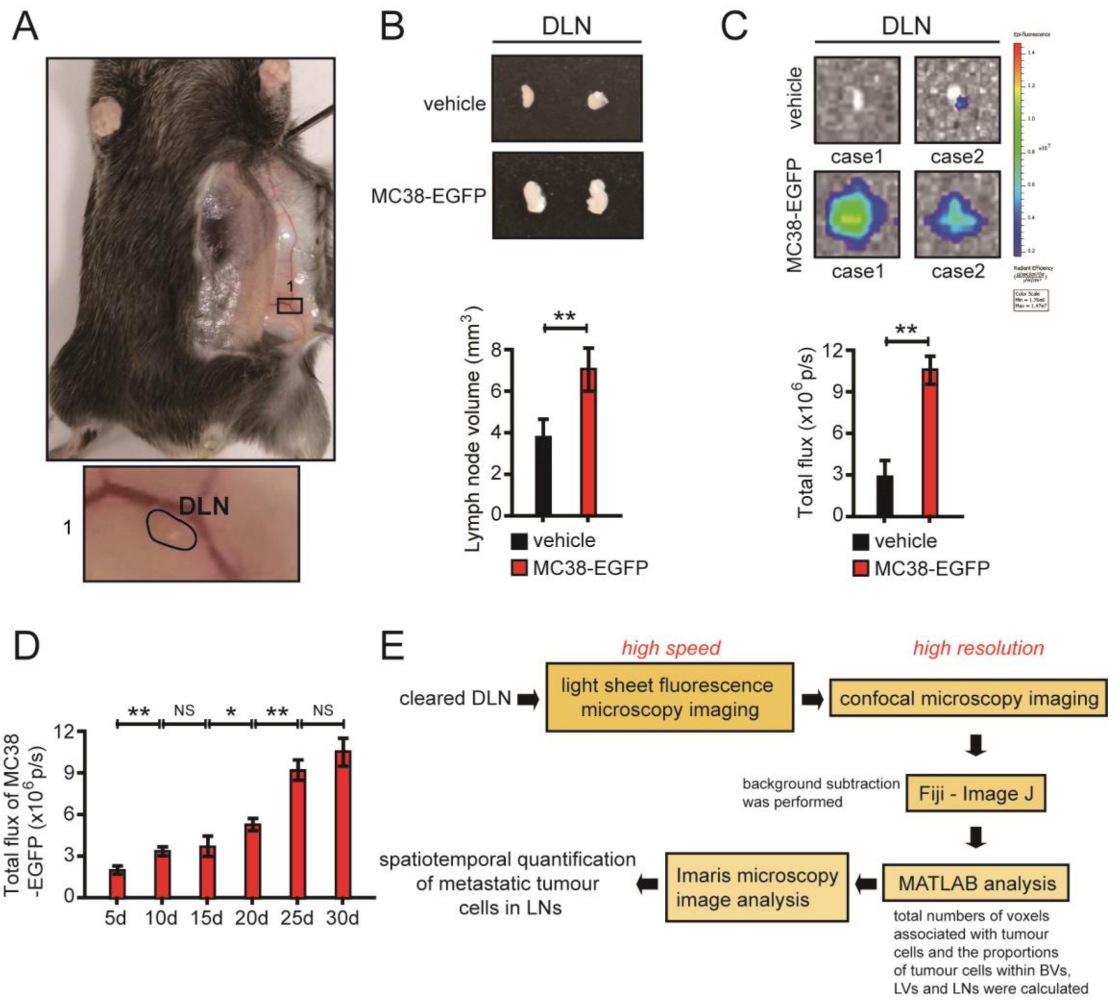

Supplementary Fig. 1. The construction of mouse footpad model.

A. Photo of DLN obtained from a C57BL/6 mouse. MC38 cells tagged with EGFP were injected into the left footpad of mice.

B. Photos of DLNs obtained from mice at the time point of 30 d. The statistical result of LN volume was shown below.

C. Fluorescent images of DLNs obtained from mice at the time point of 30 d. The statistical result of LN radiant efficiency was shown below.

D. Fluorescence intensity of EGFP in DLNs at the time points of 5 d, 10 d, 15 d, 20 d, 25 d and 30 d respectively.

E. The schematic of the detection procedure.

*: $P<0.05$; **: $P<0.01$; NS: no significant difference.

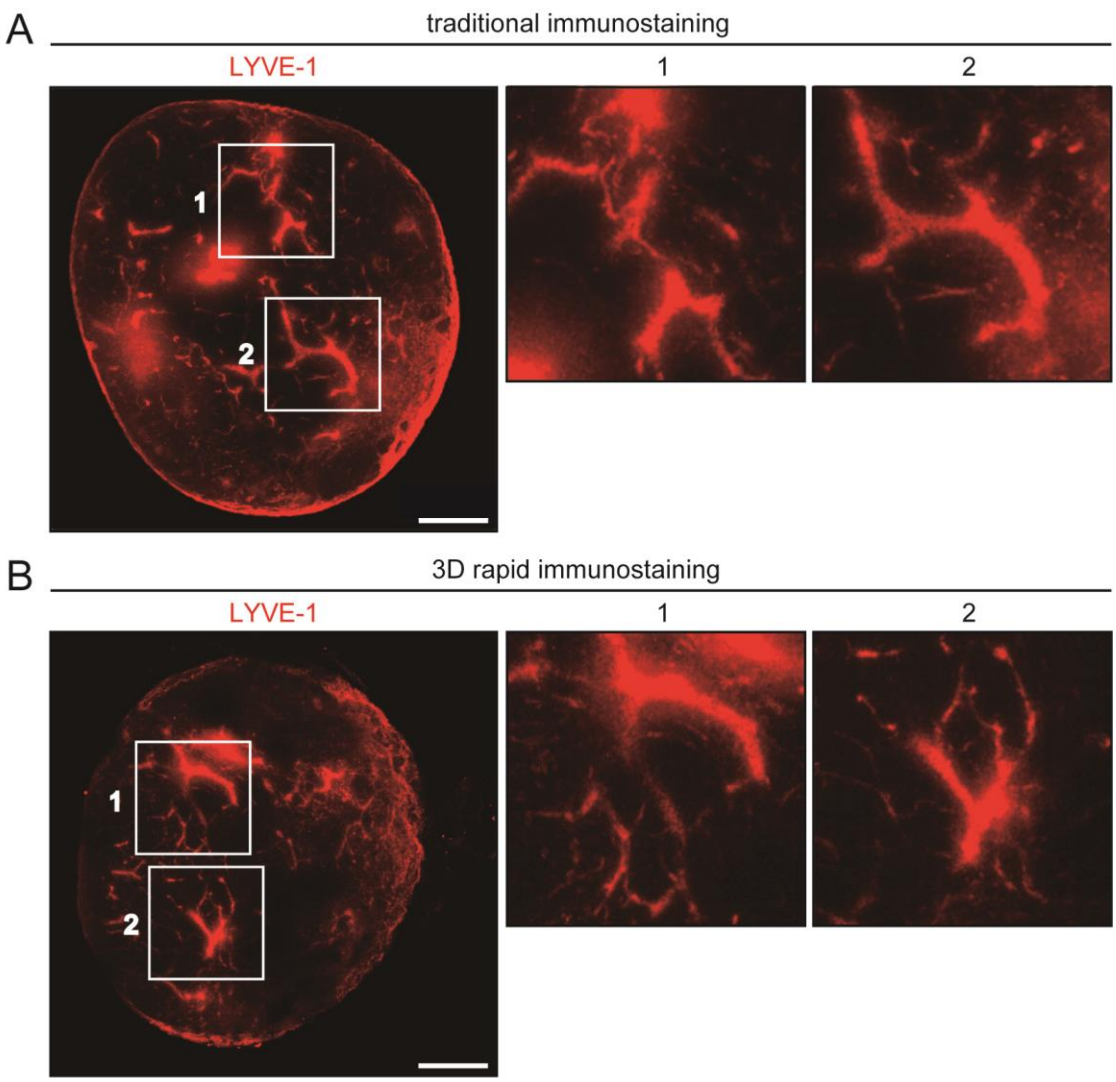

Supplementary Fig. 2. The 3D rapid immunostaining of LN.

A. Cross-section photo of LYVE-1 staining in LN treated by traditional immunostaining method.

B. Cross-section photo of LYVE-1 staining in LN treated by 3D rapid immunostaining method. Scale bars: 200 μm.

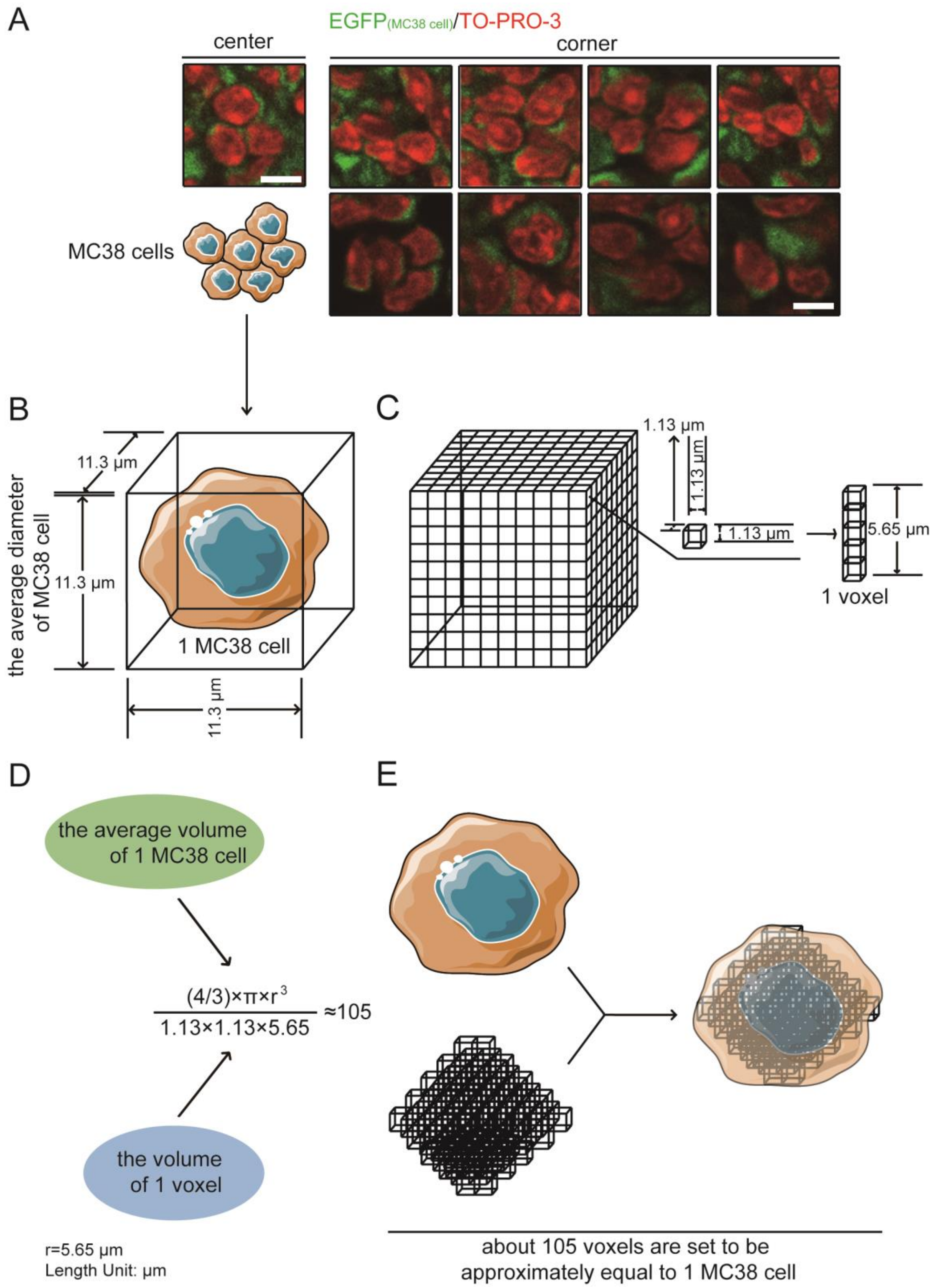

Supplementary Fig. 3. The definition of tumour cell number and tumour cell-associated voxel number.

A. The sectioning images of MC38 cells at different regions which were randomly selected. Scale bars: 10 μm..

B. The average diameter of MC38 cell that was used in this research (about 11.3 μm).

C. The definition of voxel (1.13 μm - X axis ×1.13 μm - Y axis × 5.65 μm - Z axis).

D and E. About 105 pixels are set to be approximately equal to 1 MC38 cell.

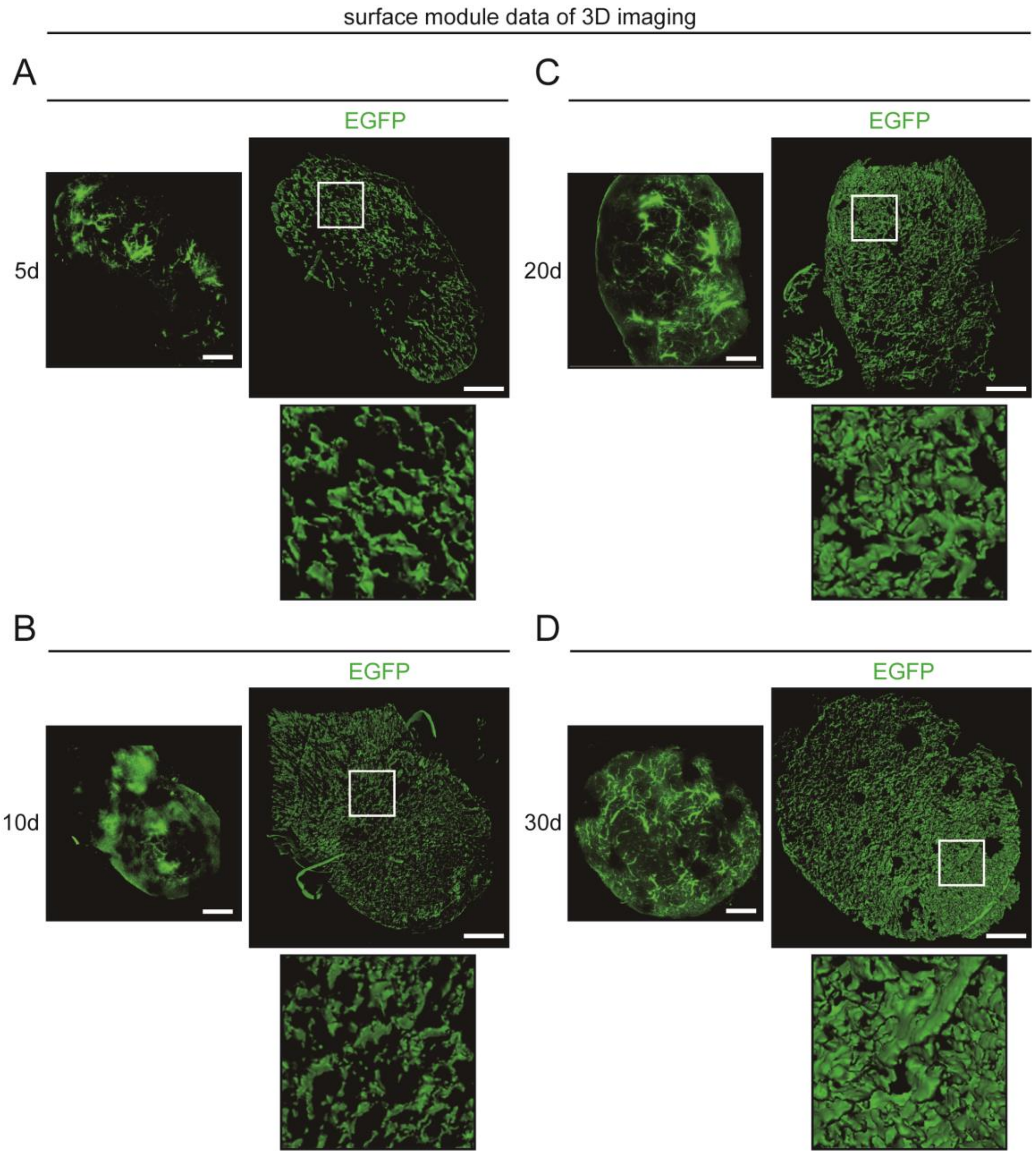

Supplementary Fig. 4. The surface module data of whole-mount tissue 3D imaging.

A-D. The surface module data of cross-section photos obtained from the 3D images of DLNs at the time points of 5 d (**A**), 10 d (**B**), 20 d (**C**) and 30 d (**D**) respectively. Scale bars: 200 μm.

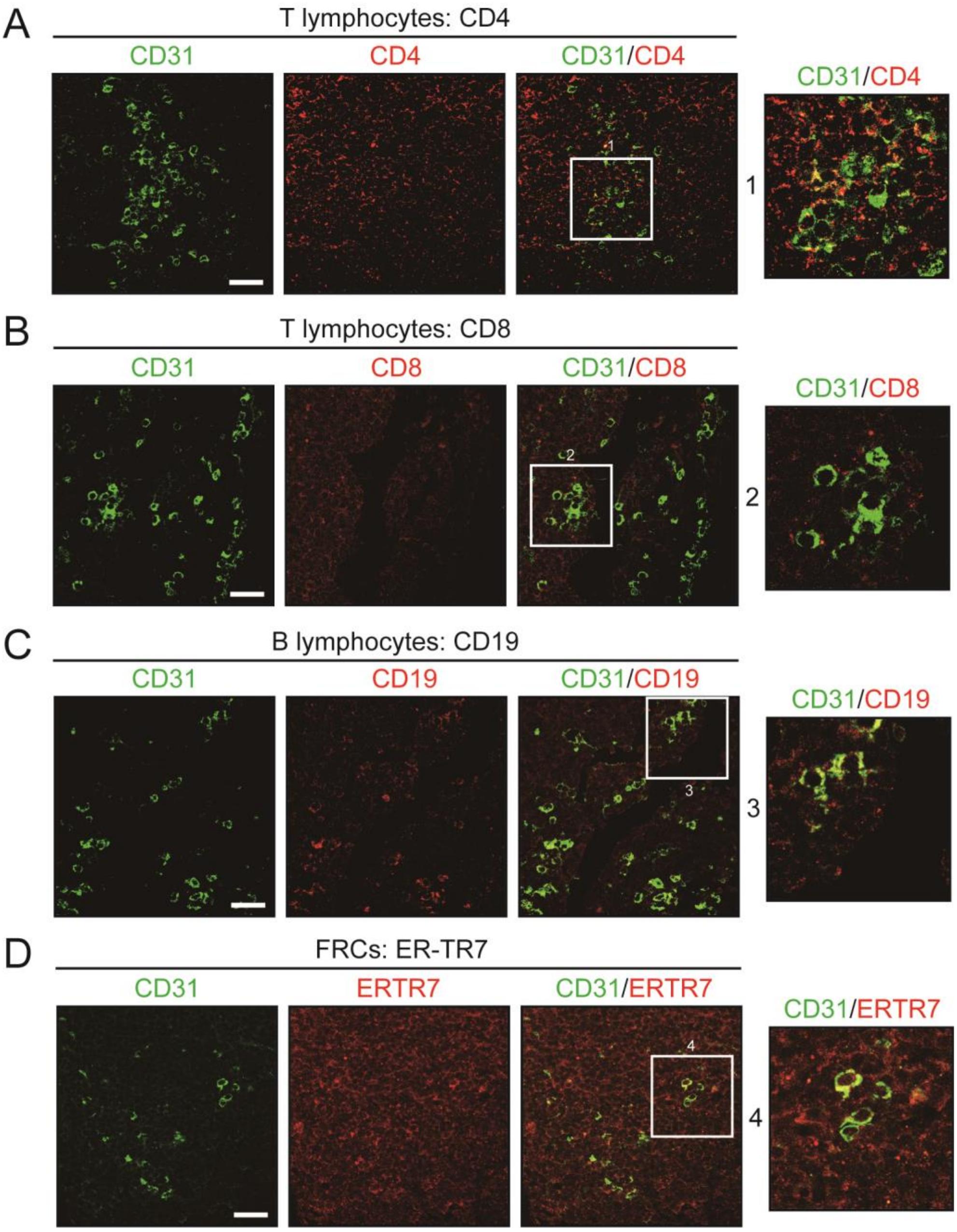

Supplementary Fig. 5. The localization of blood vessels, lymphocytes and fibroblastic reticulum cells.

A. The localization of blood vessels (CD31) and CD4+ cells (T lymphocytes).

B. The localization of blood vessels (CD31) and CD8+ cells (T lymphocytes).

C. The localization of blood vessels (CD31) and CD19+ cells (B lymphocytes).

D. The localization of blood vessels (CD31) and fibroblastic reticulum cells (FRCs).

Scale bars: 200 μm.
